# Herpes simplex virus type 2 induces S-phase entry and arrest to regulate the cell cycle for efficient replication

**DOI:** 10.1099/jgv.0.002346

**Published:** 2026-10-07

**Authors:** Huimin Wang, Ranqing Cheng, Miaomiao Li, Yuncheng Li, Siyu He, Longchao Xu, Yuhao Chen, Zhenhua Huang, Binlian Sun, Yalan Liu, Qinxue Hu, Mudan Zhang

**Affiliations:** 1 Hubei Key Laboratory of Cognitive and Affective Disorders, Institute of Biomedical Sciences, School of Medicine, Jianghan University, Wuhan 430056, PR China; 2 State Key Laboratory of Virology and Biosafety, Wuhan Institute of Virology, Chinese Academy of Sciences, Wuhan 430071, PR China; 3 Medical School, University of Chinese Academy of Sciences, Beijing 100049, PR China

**Keywords:** cell cycle, HSV-2, p21, S-phase, viral replication

## Abstract

Viruses often hijack core host cell processes to optimize their replication. In the current study, we conducted comparative proteomics analyses of epithelial cells infected by the prevalent human pathogen herpes simplex virus type 2 (HSV-2), followed by experimental validation. The results reveal that HSV-2 infection induces significant reprogramming of the host cell cycle, characterized by a pronounced accumulation of cells in the synthesis (S)-phase. Mechanistically, we discovered that HSV-2 promotes gap 1 (G1)/S transition by downregulating the cyclin-dependent kinase inhibitor p21 via a proteasome-mediated pathway. Following this, the virus induces S-phase cell cycle arrest, characterized by reduced levels of cyclin-dependent kinase 2 (CDK2) and its active phosphorylated form and decreased activity of the cyclin A2–CDK2 complex. Consequently, this dysfunctional ‘pseudo-S-phase’ state significantly enhances HSV-2 replication. These findings reveal a previously unrecognized dual mechanism employed by HSV-2 to manipulate the host cell cycle, thereby advancing our understanding of how pathogens disrupt cellular homeostasis to facilitate their replication.

## Introduction

The cell cycle is a highly regulated process in which a cell grows, replicates its DNA and divides into two daughter cells. This process is divided into four tightly coordinated phases – Gap 1 (G1), Synthesis (S), Gap 2 (G2) and Mitosis (M) [1], with differentiated cells typically remaining in a non-dividing, quiescent state (G0-phase) [2]. Re-entry into the cycle from quiescence is triggered by specific mitogenic signals [3]. The continuous progression of the cell cycle is governed by a network of checkpoint controls, cyclin-dependent kinases (CDKs), cyclin-CDK complexes and CDK inhibitors [4–8], which collectively ensure the fidelity of each phase and safeguard genomic stability [4, 5]. Consequently, dysregulation of this process is a direct threat to cell viability, leading to genomic instability and is a hallmark of cancer, degenerative diseases and cellular stress responses [9, 10].

Pathogens, especially viruses, have evolved to subvert these core regulatory networks. They typically employ various strategies to disrupt cell cycle checkpoint controls and manipulate host cell proliferation pathways, enabling efficient genome replication and progeny production [11]. For instance, many DNA or RNA viruses manipulate the host cell cycle to create a favourable environment for their replication, either by driving quiescent cells into the cycle to access nucleotide pools or by inducing arrest at a specific phase to delay host cell death and prolong the production window [12–14]. Herpesviruses are master manipulators of such cellular processes. For instance, herpes simplex virus type 1 (HSV-1) induces a G1-phase arrest [15–18], relying on its own enzymes for replication [19]. This manipulation of a basic cellular programme represents a profound disruption of cell fate.

The herpesvirus family is classified into the α-, β- and γ-herpesviruses to distinguish various biological properties, such as host range and replication kinetics [20]. As a member of the α-herpesvirus subfamily, HSV-2 is a large dsDNA virus and the primary causative agent of genital herpes. Predominantly transmitted through sexual contact, HSV-2 infected an estimated 519.5 million individuals aged 15–49 years globally by 2020, representing a substantial public health burden [21, 22]. HSV-2 is highly transmissible and characterized by lifelong symptomatic recurrence, with potential severe complications, including neonatal herpes and meningitis [23].

While the impact of herpesviruses on cell cycle regulation has been explored [14, 17, 24–26], the specific relationship between HSV-2 infection and cell cycle modulation remains unclear to date. Despite its close relation to HSV-1, preliminary evidence suggests a divergent strategy in cell cycle manipulation. We previously reported that the HSV-2 late protein UL24 promotes an increased proportion of cells in S-phase [27]. Given that S-phase is a tightly regulated, energy-intensive process characterized by a rich pool of nucleotides and replication machinery [28–30], viruses often exploit this cell process to enhance their own replication [14]. Therefore, this study aims to investigate how HSV-2 interfaces with the host’s core cell cycle machinery and to what functional end.

In the current study, we demonstrate that HSV-2 drives cells into S-phase by triggering the proteasome-mediated degradation of the key cyclin-dependent kinase inhibitor (CKI) p21 and subsequently induces a functional intra-S-phase arrest by affecting the core S-phase regulators (CDK2, cyclin A2–CDK2 complex). This creates a ‘pseudo-S-phase’ state, characterized by a resource-rich yet replication-incompetent cellular environment, which favours viral replication while hindering normal cell cycle progression.

## Methods

### Cells and viruses

The human cervical epithelial cell line (HeLa), African green monkey kidney cell line Vero, human foreskin fibroblasts cell line (HFF-1) and human embryonic kidney cell line HEK 293T were maintained in Dulbecco’s Modified Eagle Medium (DMEM) (C11995500BT; Life Technologies, Carlsbad, CA, USA) supplemented with 10% FBS (10099–141; Gibco, New York, NY, USA), 100 U ml^−1^ penicillin and 100 U ml^−1^ streptomycin at 37 °C in a 5% CO_2_ humidified incubator. The spontaneously arising retinal pigment epithelia (RPE) cell line ARPE-19 was maintained in DMEM/Nutrient Mixture F-12 (C11330500BT; Life Technologies) supplemented with 10% FBS, 100 U ml^−1^ penicillin and 100 U ml^−1^ streptomycin at 37 °C in 5% CO_2_. GFP-labelled HSV-2 (186 strain) described in previous studies [31, 32] was propagated in Vero cells and titrated by plaque assay on confluent Vero monolayers. Aliquots of the virus stock were stored at −80 °C supplemented with 10% FBS.

### Antibodies and drugs

Antibodies (Abs) against cyclin A2, CDK2 and β-actin were purchased from Proteintech (18202-1-AP, 10122-1-AP and 66009-1-Ig; Wuhan, China). Phospho-CDK2-T160 Ab was from ABclonal Technology (AP1364; MA, USA). Ab against human p21 was purchased from Selleck (F0170; Houston, USA). Ab against Flag tag was purchased from Sigma-Aldrich (F1804; Saint Louis, MO, USA). Abs against HSV-2 and E2F3 were from Abcam (ab21112 and ab320731; MA, USA). HRP-conjugated Goat anti-Rabbit (SA00001-2; Proteintech, Wuhan, China), Goat anti-Mouse (SA00001-1; Proteintech, Wuhan, China) and Rabbit anti-Goat (BA1060; Boster, Wuhan, China) IgG were used as secondary Abs for Western blot analysis. The autophagy inhibitor Spautin-1 (SC5498) and proteasome inhibitor Bortezomib (SC0263) were purchased from Beyotime Biotechnology (Shanghai, China).

### Plasmids

The construction of Flag-tagged expression plasmids for HSV-2 UL54, RL2, RS1, US1, US12 and UL24 has been previously described in our studies [27, 31]. For the p21 expression plasmid, cDNA derived from HeLa cells was used as a template for PCR amplification. A C-terminal Flag tag was incorporated into the amplicon via the reverse primer. The vector pcDNA3.1(+) (Invitrogen) was digested with HindIII and NotI (R3104S and R3189S; New England Biolabs, MA, USA) and subsequently ligated with the p21 cDNA through homology recombination. The constructed p21 expression plasmid was designated as p21-Flag. All primers used for plasmid construction are listed in Table S1.

### The titration of HSV-2

HSV-2 viral titres were determined by plaque assays. Briefly, confluent monolayers of Vero cells were infected with serially diluted HSV-2. Following a 1 h adsorption period, the inoculum was removed and the cells were maintained in medium containing 2% (v/v) FBS and 1% (wt/v) methylcellulose. At 48 h post-infection (hpi), the cells were fixed and stained with crystal violet. Subsequently, the number of visible plaques was counted to calculate the viral titre, expressed as p.f.u. per millilitre (p.f.u. ml^−1^).

### Proteomic analysis by mass spectrometry

### Sample preparation

HeLa cells infected with HSV-2 (m.o.i.=3) at 1, 8 and 16 hpi were lysed in RIPA buffer supplemented with a protease inhibitor cocktail (11697498001; Roche, Mannheim, Germany) on ice for 30 min. Lysates were centrifuged at 12,000×*g* for 10 min at 4 °C to remove cellular debris. The supernatant was collected, and protein concentration was determined using an Enhanced BCA Protein Assay Kit (P0010S; Beyotime, Shanghai, China). For each biological replicate (*n*=3 per condition), 40 µg of protein was subjected to acetone precipitation and subsequent tryptic digestion for mass spectrometric analysis.

### Liquid chromatography and mass spectrometry

Liquid chromatography and mass spectrometry analysis was performed by Shanghai Biotree Co., Ltd. Briefly, digested peptides were separated on an EASY-Spray™ HPLC reversed-phase column (Thermo Scientific, IL, USA) using a Vanquish neo UHPLC system (Thermo Scientific, USA), and then analysed online with an Astral mass spectrometer (Thermo Scientific, USA) equipped with a nano-electrospray ion source. Mass spectrometry data were acquired in data-independent acquisition (DIA) mode with the following settings: MS1 resolution=240K, mass range=380–980 m/z; MS2 mass range=150–2,000 m/z; DIA scans were performed with 2 m/z isolation windows.

### Bioinformatics processing

Raw DIA data were processed using MaxQuant software (v2.0.3) and searched against the UniProt Homo sapiens reference proteome database (release 2024_05). Carbamidomethylation of cysteine was set as a fixed modification, while methionine oxidation and N-terminal acetylation were specified as variable modifications. The false discovery rate (FDR) for peptide and protein identification was set to 1%. A total of 9,185 protein groups were identified and quantified across all samples. Differential expression analysis was performed using a Student’s t-test, while proteins with *P*<0.05 and fold change ≤0.83 or ≥1.2 were considered statistically and biologically significant. Functional enrichment analysis of KEGG pathways was performed in R using ggplot2 (v3.3.2), with pathways having an FDR <0.05 considered significantly enriched. Visualization was performed in R using ggplot2 (v3.3.2) for volcano plots and pheatmap (v1.0.12) for hierarchical clustering analysis.

### Cell cycle analysis

Cell cycle distribution was analysed using the Cell cycle Analysis Kit (C1052; Beyotime, Shanghai, China). Briefly, cells were transfected with an empty vector or Flag-tagged UL54, RL2, RS1, US1, US12 or UL24 expression plasmids for 48 h or infected with HSV-2 for 24 h. Subsequently, the cells were fixed in 70% ethanol overnight at 4 ℃. After a wash with 1× PBS, the cells were resuspended in a PI/RNase Staining Buffer and incubated for 30 min at 37 ℃ in the dark. Samples were then measured on a BD LSRFortessa flow cytometer (Piscataway, NJ, USA), and the cell cycle profiles of 1×10^4^ events per sample were analysed using ModFit LT 5.0 software (Verity Software House, ME, USA). The percentages of cells in each cell cycle phase were analysed using GraphPad Prism 9.

### Cell cycle synchronization

Vero and HFF-1 cells were seeded in 6-well plates and cultured in complete growth medium overnight reaching ∼50% confluence. To induce cell cycle synchronization, Vero and HFF-1 cells were then cultured in serum-free DMEM for 36 h or 48 h, and synchronization efficiency was confirmed by flow cytometry. These synchronized cells were inoculated with HSV-2 at an m.o.i. of 3 for 1 h. After the adsorption period, the inoculum was removed and replaced with fresh complete medium DMEM. At 6 hpi or 12 hpi, the total virus was collected for titre determination by plaque assay.

### Western blot analysis

Western blotting was performed as previously described [31, 32]. Briefly, cells were transfected with the indicated plasmid for 48 h or infected with HSV-2 for 24 h. Cells were collected and resuspended in lysis buffer (P0013J; Beyotime, Shanghai, China) with protease inhibitor cocktail (HY-K0010; MedChemExpress, Shanghai, China), followed by incubation on ice for 10 min. Lysates were then centrifuged at 12,000 *g* for 10 min at 4 °C, and then the supernatants were collected and mixed with 5× SDS sample loading buffer. Proteins were separated by electrophoresis on FuturePAGE™ 4–20% gradient gel (ET15420; ACE Biotechnology, Changzhou, China) and transferred to a PVDF membrane (IPVH00010; Millipore, Darmstadt, Germany). After blocking, membranes were incubated with the designated primary Abs. Protein bands were visualized using the ChemiScope System (Clinx, Shanghai, China) with an enhanced chemiluminescent substrate (K-12045-D50; Advansta, CA, USA). β-actin was used as a loading control. Protein molecular weight marker was from Thermo Scientific (26616; IL, USA).

### Quantitative real-time PCR

Total RNA was extracted using the RNAeasy™ Isolation Kit (R0026; Beyotime, Shanghai, China) and then reverse-transcribed into cDNA with HiScript II Q RT SuperMix (R223; Vazyme, Nanjing, China) according to the manufacturer’s instructions. qPCR was performed with Taq Pro Universal SYBR qPCR Master Mix (Q712; Vazyme, Nanjing, China) on a Bio-Rad CFX Connect Real-time System under the following conditions: 95 °C for 30 s, followed by 40 cycles of 95 °C for 10 s and 60 °C for 30 s. A melting curve analysis was conducted from 65 to 95 °C with 0.5 °C increments. Primer sequences are listed in Table S1. Relative gene expression was calculated on the basis of 2^−ΔΔCt^ values.

### The detection of cyclin A2–CDK2 activity

To determine the kinase activity of cyclin A2–CDK2 complexes, HeLa cells were seeded in 10 cm dishes and cultured for 24 h. Subsequently, cells were infected with HSV-2 at an m.o.i. of 0.05 for an additional 24 h. At the indicated time points, cells were collected, lysed and total protein was extracted. Kinase activity was determined using a commercial cyclin A–CDK2 kinase detection kit (GMS50146.1; Genmed, Shanghai, China) according to the manufacturer’s protocol. Briefly, 130 µl of assay buffer, 20 µl of enzyme mix, 20 µl of reaction solution and 20 µl of substrate solution were sequentially added into the wells of a 96-well plate. The mixture was gently shaken and pre-incubated at 30 °C for 3 min. Subsequently, 10 µl of either the negative control or the test sample containing 100 µg of cell lysate protein was added to the respective wells. The absorbance at 340 nm was then repeatedly measured at intervals within 5 min using a Multimode Plate Reader (EnSpire, PerkinElmer). The cyclin A2–CDK2 kinase activity was calculated based on the difference between the initial and final absorbance readings at 340 nm (ΔOD_₃₄₀_) according to the formula provided by the manufacturer.

### Statistical analysis

Data are presented as mean±sd from at least three independent experiments, each performed in duplicate or triplicate unless otherwise specified. Statistical analyses were conducted using GraphPad Prism 9 (GraphPad). Differences between two groups were assessed by an unpaired two-tailed Student’s *t*-test, and multiple group comparisons were analysed by one-way or two-way ANOVA with Tukey’s test. *P*<0.05 was considered statistically significant.

## Results

### Proteomic profiling implicates host cell reprogramming by HSV-2

Previous studies have demonstrated that viruses, including herpesviruses, manipulate the host cell cycle to facilitate their replication [11, 14, 33, 34]. However, different herpesviruses may have evolved specific strategies to manipulate host cells, as suggested by our previous studies [31, 32]. To explore how HSV-2, a pathogen that primarily targets genital epithelial cells, influences cell cycle progression, we conducted quantitative proteomic analysis of HSV-2-infected HeLa cells, a widely used cervical epithelial cell model for HSV-2 infection, at 1, 8 and 16 hpi. Hierarchical clustering analysis of differentially expressed proteins revealed high reproducibility across three biological replicates and clear separation between the HSV-2-infected and mock-infected groups, confirming a robust host response (Figs 1a–b and S1A). Over time, HSV-2 infection led to a progressive increase in the number of dysregulated proteins (Fig. S1A, Fig. 1a–c). Notably, KEGG pathway enrichment analysis revealed a strong association between the differentially expressed proteins and the cell cycle pathway at 16 hpi (Figs 1d–e and S1B). Taken together, these findings provide initial evidence that HSV-2 infection directly and potently targets the host cell cycle, signifying a disruption of cell cycle homeostasis.

**Fig. 1.**
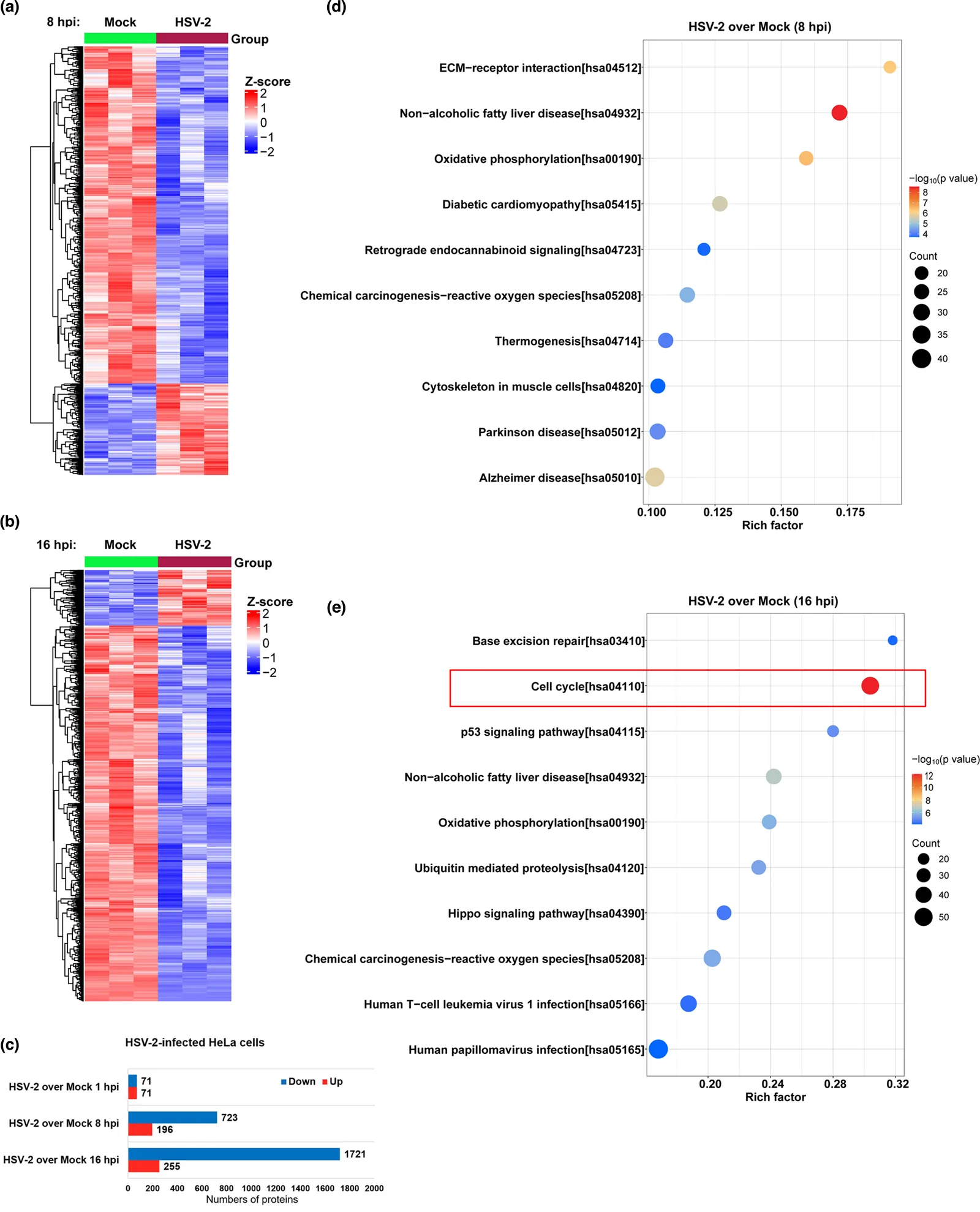
Proteome profiling reveals that HSV-2 infection modulates cell cycle progression. (**a-b**) Hierarchical clustering analysis of differentially expressed proteins in HSV-2-infected HeLa cells at the indicated time points. Each column represents an individual biological replicate, and each row represents a protein. Expression levels are Z-score normalized (red, up-regulated; blue, down-regulated). (**c**) Bar plot summarizing the number of significantly up- and down-regulated proteins at each time point. (**d-e**) KEGG pathway enrichment analysis of the differentially expressed proteins at the indicated time points. The size of the dots corresponds to the number of proteins mapped to each pathway, while the colour represents the −log_10_ (p value).

### HSV-2 infection drives S-phase accumulation

To further validate the proteome analysis, we examined the effect of HSV-2 on cell cycle in HeLa cells, a widely used cervical epithelial cell model for HSV-2 infection. Infection with HSV-2 (m.o.i.=0.01 or 0.05) for 24 h led to an obvious accumulation of cells in the S-phase, as quantified by flow cytometry (Fig. 2a–c). To further confirm our findings, we repeated the experiments in Vero cells, a commonly used cell line for HSV-2 propagation. Consistent with the results in HeLa cells, HSV-2 infection also induced S-phase accumulation in Vero cells (Fig. 2d–f). Furthermore, we observed that HSV-2 infection notably reduced the percentages of cells in the G1-phase while increasing the proportions in S-phase (Fig. 2b and e), indicating an enhanced G1/S transition as previously described [35–37]. To further validate our findings in an untransformed cell model permissive to HSV-2 infection, we employed HFF-1 cells, a cell line retaining WT p53 and Rb expression and exhibiting normal cell cycle regulation [38, 39]. Notably, findings from HFF-1 cells were consistent with those obtained from HeLa and Vero cells (Fig. 2g–i), suggesting that HSV-2-induced cell-cycle modulation represents a conserved response that is not limited to transformed or immortalized cell lines. Collectively, these results indicated that HSV-2 infection drives S-phase accumulation by inducing cells into S-phase.

**Fig. 2.**
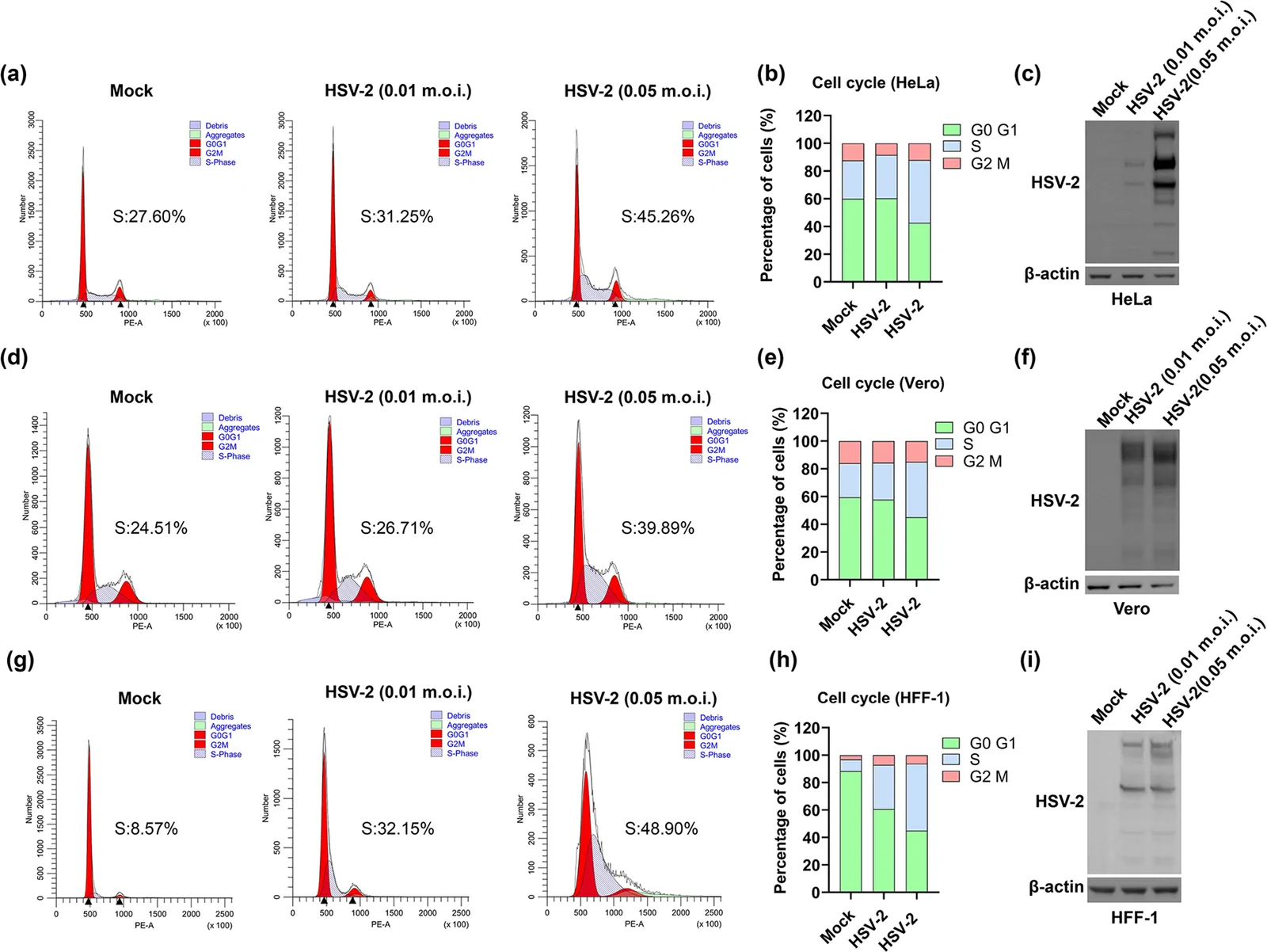
HSV-2 drives S-phase accumulation by enhancing G1/S transition. HeLa, Vero or HFF-1 cells were infected with HSV-2 at the indicated dose. At 24 hpi, cells were harvested to analyse the cell cycle distribution by flow cytometry analysis (**a, d and g**) or lysed for Western blot assay to confirm successful infection (**c, f and i**). The percentages of cells in each cell cycle phase were analysed using GraphPad Prism 9 (**b, e and h**). Data shown are representative of three independent experiments.

### HSV-2 promotes S-phase entry through proteasomal degradation of p21

To investigate the mechanism underlying HSV-2-mediated G1/S phase transition, we re-analysed these differentially expressed proteins in the proteomic profile. The volcano plots revealed that HSV-2 infection induced widespread differential protein expression, with both the number of differentially expressed proteins and the fold changes increasing over time (Fig. 3a–c). Among these proteins, we focused on p21, a CKI and key negative regulator of S-phase entry, which was notably downregulated in the cell cycle signalling pathway at 16 h post-HSV-2 infection (Fig. 3c). p21, encoded by cyclin-dependent kinase inhibitor 1A (*CDKN1A*), is the first identified and characterized member of the CKI family [40]. It blocks the transition of cell cycle from the G1-phase to S-phase by directly inhibiting the cyclin E–CDK2 complex [40–42]. Based on this, we hypothesize that p21 is a critical target in HSV-2-induced S-phase accumulation.

**Fig. 3.**
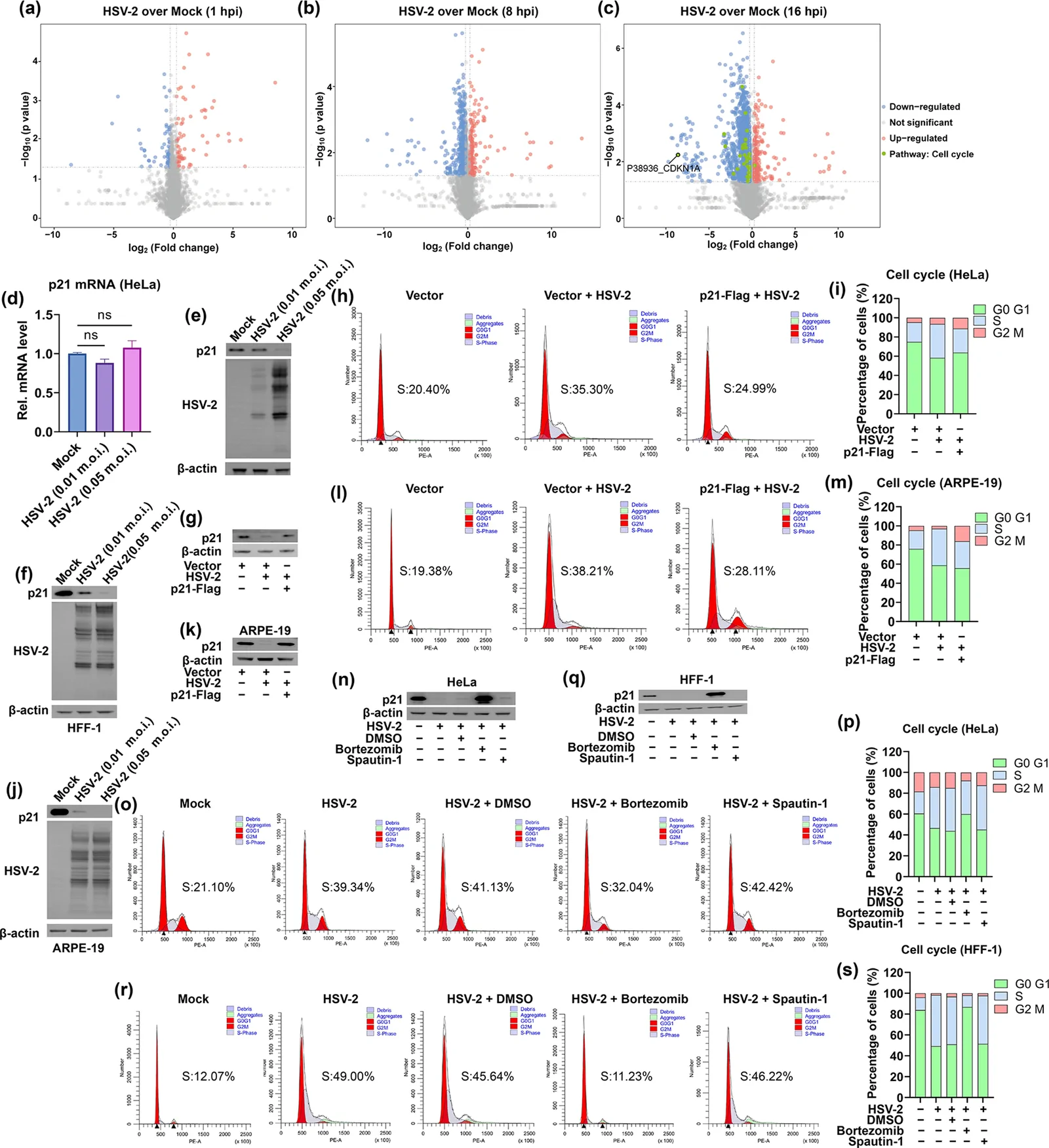
HSV-2 promotes S-phase entry through proteasomal degradation of p21. (**a-c**) Volcano plot of proteomic data revealed that p21 is a pivotal target in HSV-2-induced S-phase accumulation. (**d-f and j**) HSV-2 infection induced the decrease of p21 at the protein level. HeLa (**d-e**), HFF-1 (**f**) or ARPE-19 (**j**) cells were infected with HSV-2. At 24 hpi, the mRNA and protein levels of p21 were measured by qPCR (**d**) and Western blot (**e-f and j**), respectively. (**g-m**) Restoration of p21 weakened S-phase accumulation mediated by HSV-2 infection. HeLa (**g-i**) or ARPE-19 (**k-m**) cells were transfected with empty vector or p21 expression plasmid for 24 h, followed by infection with HSV-2. At 24 hpi, the restoration of p21 was detected by Western blot (**g and k**), while the cell cycle was analysed by flow cytometry analysis (**h and l**). (**n and q**) HSV-2 induced the proteasome-mediated degradation of p21. (**o-p**) and (**r-s**) Proteasome inhibition largely attenuated HSV-2-induced S-phase accumulation. HeLa (**o-p**) or HFF-1 (**r-s**) cells were infected with HSV-2 (m.o.i.=0.05) and treated with DMSO, the proteasome inhibitor Bortezomib (10 µM) or the autophagy inhibitor Spautin-1 (10 µM) from 1 to 24 hpi. p21 levels were assessed by Western blot (**n and q**), while the cell cycle was analysed by flow cytometry analysis (**o and r**). The percentages of cells in each cell cycle phase were analysed (i, m, p and s). ns, not significant. Data shown are representative of three independent experiments.

To elucidate the mechanism by which HSV-2 promotes S-phase accumulation, we first assessed the expression of p21 in HSV-2-infected HeLa cells. While p21 mRNA levels showed no significant change (Fig. 3d), p21 protein was substantially depleted (Fig. 3e), suggesting post-transcriptional regulation of p21 in HSV-2 infection. This observation was independently validated in untransformed HFF-1 cells (Fig. 3f). Crucially, re-establishing p21 expression via transfection effectively reversed HSV-2-induced G1/S phase transition and S-phase accumulation in HeLa cells (Fig. 3g–i). Given the low transfection efficiency of HFF-1 cells [43–45], we further performed mechanistic studies under the condition of transfection in ARPE-19 cells, an untransformed RPE line that retains WT Rb and p53 expression [46] and is permissive to HSV-2 infection. In ARPE-19 cells, HSV-2 infection similarly induced S-phase accumulation (Fig. S2A-C) and the depletion of p21 protein (Fig. 3j), further confirming our observations in HeLa and HFF-1 cells. Importantly, the efficient plasmid transfection capacity of ARPE-19 cells enabled functional rescue experiments, demonstrating that p21 restoration effectively reversed HSV-2-induced cell cycle alterations (Fig. 3k–m). Together, these results establish that HSV-2 promotes S-phase entry through downregulation of the CKI p21.

Given that p21 expression was unchanged at the mRNA level, we hypothesize that p21 is likely degraded at the protein level. The ubiquitin-proteasome system (UPS) and autophagy are two major intracellular protein degradation pathways in eukaryotic cells [47, 48]. The UPS typically degrades the majority of proteins, while autophagy mainly clears long-lived or aggregated components [49]. To determine the mechanism underlying HSV-2-mediated p21 downregulation, HSV-2-infected HeLa or HFF-1 cells were treated with the proteasome inhibitor Bortezomib or the autophagy inhibitor Spautin-1. As shown in Fig. 3n and q, Bortezomib, but not Spautin-1, markedly restored p21 levels in HSV-2-infected cells, suggesting that HSV-2 induces p21 degradation via the proteasome. Consistently, the HSV-2-induced G1/S phase transition was largely attenuated when p21 degradation was inhibited by Bortezomib (Fig. 3o–p and r–s). Collectively, these results demonstrate that HSV-2 promotes S-phase entry through proteasomal degradation of p21.

### HSV-2 induces intra-S-phase arrest

Based on the above observations, we conclude that HSV-2 can promote S-phase entry through proteasomal degradation of p21, which predicts an eventual increase in cell number. Interestingly, we found that the number of cells did not increase but instead significantly decreased after HSV-2 infection (Fig. 4a–b), suggesting that HSV-2 likely suppresses the normal progression of the S-phase and traps the cells in this phase. The cyclin A2–CDK2 complex is a key regulator of the cell cycle, acting both as the ‘engine’ driving S-phase progression and as the ‘timer’ ensuring the orderly and irreversible execution [50–53]. To test our hypothesis, we first measured the protein levels of cyclin A2 and CDK2. The results showed that HSV-2 infection markedly reduced the activity of the cyclin A2–CDK2 complex (Fig. 4c) in HeLa and the levels of CDK2 and phospho-CDK2 in HeLa, HFF-1 and ARPE-19 cells, accompanied by decreased cyclin A2 abundance in HFF-1 and ARPE-19 cells (Fig. 4d–f). KEGG pathway enrichment analysis of the proteome profile further revealed that HSV-2 infection profoundly disrupted pathways associated with the host DNA replication machinery. This was exemplified by the significant enrichment of downregulated proteins within essential DNA replication complexes, including DNA polymerase, helicase, RNase and clamp loader complexes, all of which are critical for DNA synthesis and replication fork progression (Fig. 4g). Together, these results indicate that HSV-2 not only promotes S-phase entry but also induces an intra-S-phase arrest.

**Fig. 4.**
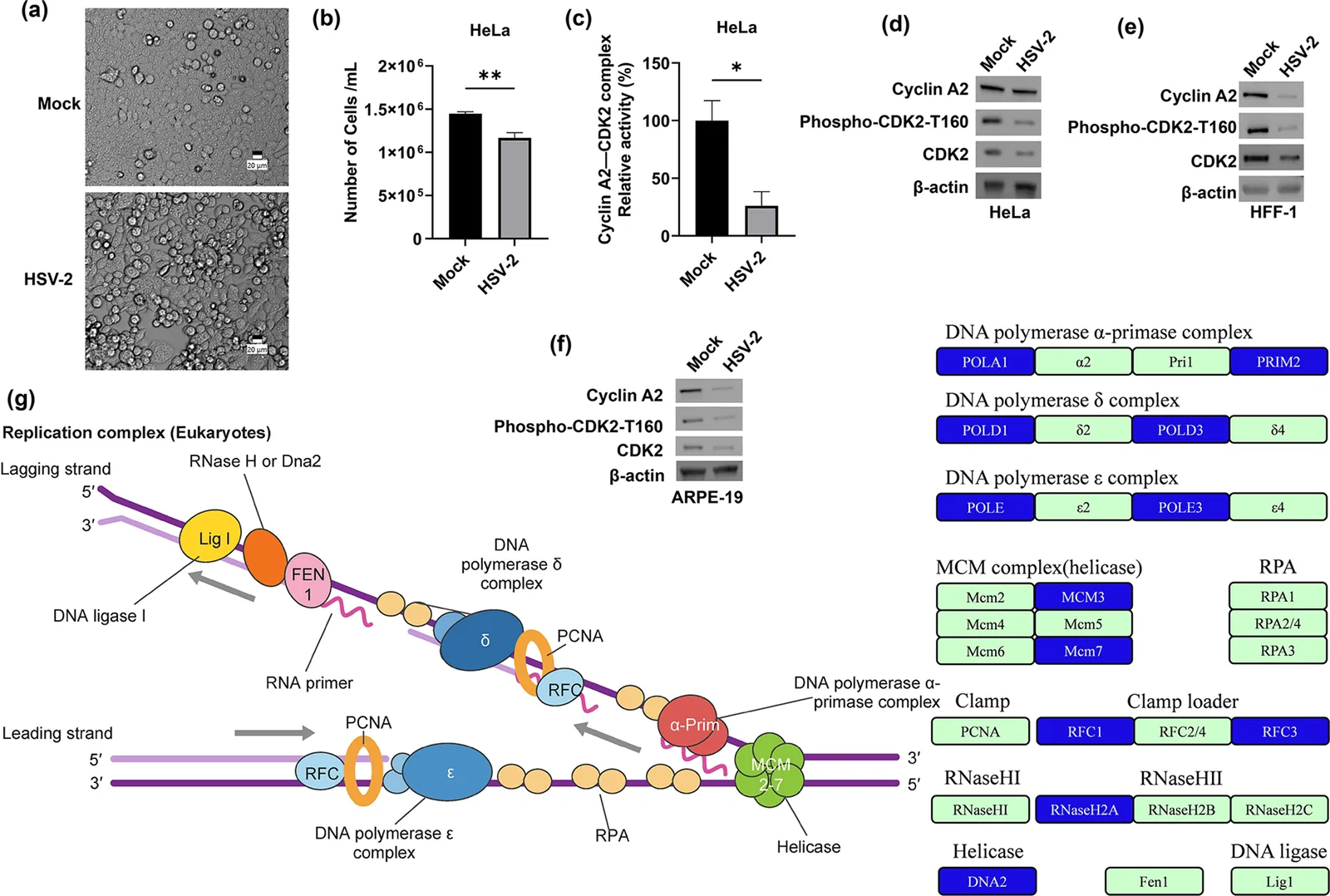
HSV-2 induces cell cycle arrest in S-phase. (**a-b**) HSV-2 infection induced the decrease of HeLa cell number. (**c**) The activity of the cyclin A2–CDK2 complex decreased in HSV-2-infected HeLa cells. (**d-f**) HSV-2 induces the dysregulation of key S-phase regulatory proteins. HeLa (**a-d**), HFF-1 (**e**) or ARPE-19 (**f**) cells were infected with HSV-2 (m.o.i.=0.05). At 24 hpi, the HeLa cell images were captured using an Olympus microscope (Olympus, Tokyo, Japan) and HeLa cell numbers were determined using a TC20™ Automatic Cell Counter (Bio-Rad, CA, USA) (**a-b**). To determine the kinase activity of the cyclin A2–CDK2 complex, HeLa cells were infected with HSV-2 (m.o.i.=0.05). At 24 hpi, cell lysates were prepared, and kinase activity was measured with a Multimode Plate Reader (**c**). The protein levels of cyclin A2, phospho-CDK2-T160 and CDK2 were assessed by Western blot using specific primary Abs (**d-f**). (**g**) Proteome profiling revealed that HSV-2 infection dysregulated the host cell DNA replication. Proteins are grouped by functional modules involved in the replication process. Downregulated proteins are marked in blue, while proteins with non-significant changes are shown in green. *P<0.05, **P<0.01. Data shown are representative of three independent experiments.

### HSV-2-induced S-phase accumulation creates a favourable environment for viral replication

The S-phase is usually characterized by the upregulation of host DNA replication machinery, including DNA polymerases, replication cofactors, histones and enzymes involved in nucleotide synthesis [54–56]. In our study, HSV-2 infection induced an intra-S-phase arrest, while the proteome profile further revealed that HSV-2 infection profoundly disrupted pathways associated with the host DNA replication machinery. It is known that HSV-2 encodes its own nucleotide metabolism enzymes, such as ribonucleotide reductase and thymidine kinase [19]. These together raise the question of whether HSV-2 takes advantage of the S-phase environment it induces to promote its replication. It is well established that in the absence of growth factors, a cell will exit the cell cycle and enter the G0-phase [57, 58], while the re-addition of serum or growth factors typically drives cells back into the G1-phase, resuming normal cycle progression [59, 60]. Next, we synchronized Vero and HFF-1 cells through serum starvation, which reduced the S-phase population (Fig. 5a–b and d–e) and concurrently decreased HSV-2 titres (Fig. 5c and f), indicating that the S-phase is favourable for HSV-2. In contrast, restoring p21 – whose downregulation is essential for S-phase entry– significantly impaired HSV-2 replication in both HeLa and ARPE-19 cells (Fig. 5g–j). Furthermore, inhibition of the proteasome, but not autophagy, restored the protein level of p21 and attenuated HSV-2 replication in both HeLa and HFF-1 cells (Fig. 5k–n), supporting the link between p21 degradation and efficient HSV-2 production. Collectively, these results demonstrate that HSV-2-induced reprogramming of the host cell cycle into a ‘pseudo-S-phase’ creates a cellular state conducive to viral replication.

**Fig. 5.**
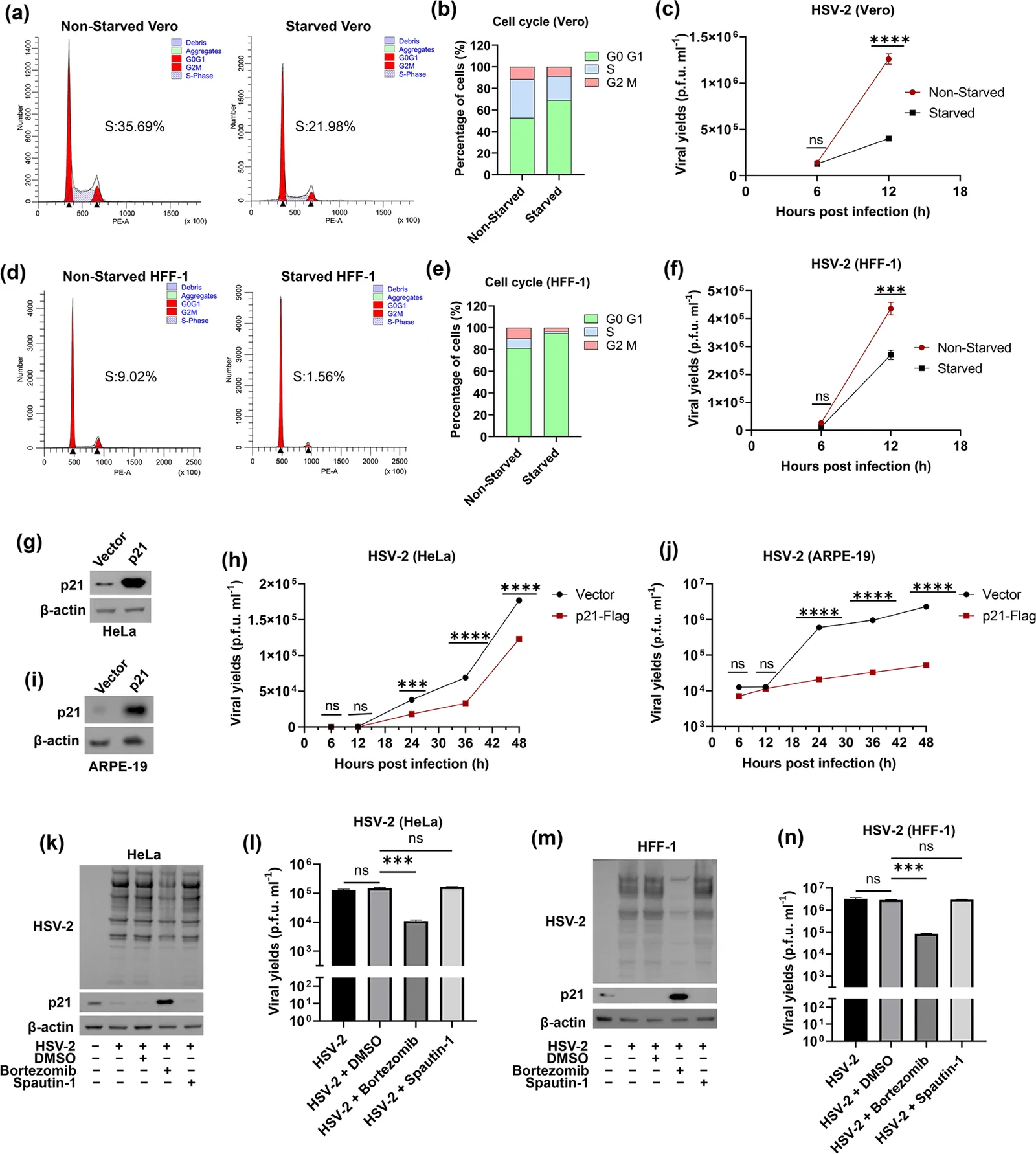
HSV-2-induced S-phase accumulation creates a favourable environment for viral replication.(**a-b**) and (**d-e**) The percentages of cells in S-phase decreased under serum starvation. Vero or HFF-1 cells were cultured in serum-free medium for 36 h or 48 h. The cell cycle was analysed by flow cytometry analysis (**a and d**). The percentages of cells in each cell cycle phase were analysed (**b and e**). (**c and f**) The yield of HSV-2 in starved cells was significantly decreased. Vero or HFF-1 cells were cultured in serum-free medium for 36 h or 48 h, followed by infection with HSV-2 (m.o.i.=3). The total HSV-2 was collected at different time points following infection and titrated by plaque assay. (**g-j**) Restoration of p21 significantly attenuated the increase yield of HSV-2. HeLa or ARPE-19 cells were transfected with an empty vector or a p21 expression plasmid for 24 h, followed by infection with HSV-2 (m.o.i.=0.01). HSV-2 was collected at different time points following infection and titrated by plaque assay (**h and j**). The expression of p21 at 48 hpi was detected using anti-p21 Ab (**g and i**). (**k-n**) The yield of HSV-2 significantly decreased under the inhibition of p21 degradation. HeLa or HFF-1 cells were infected with HSV-2 (m.o.i.=0.05) and treated with DMSO, Bortezomib (10 µM) or Spautin-1 (10 µM) at 1 hpi. At 24 hpi, HSV-2 infection was assessed by Western blot (**k and m**), and total viral yields were quantified by plaque assay (**l and n**). ***P<0.001, ****P<0.0001, ns, not significant. Data shown are representative of three independent experiments.

## Discussion

The cell cycle is a fundamental biological process that governs controlled cell growth, DNA replication and division. Its precise regulation is crucial for maintaining genomic stability, tissue homeostasis and preventing diseases, such as cancer [61, 62]. The cell cycle is essential for cellular homeostasis and serves as a central battlefield for host–virus interactions. For viruses, particularly DNA viruses, the cell cycle represents a strategic target for hijacking host resources to facilitate viral replication. Understanding how viruses manipulate cell cycle regulation could offer valuable insights into pathogenesis, antiviral therapy and cancer biology.

In this study, we found that HSV-2 promotes S-phase entry by degrading the CDK inhibitor p21. In contrast to other herpesviruses, such as HSV-1, HCMV and EBV, which induce G0/G1 arrest during lytic infection [17, 63–66], HSV-2 drives G1/S phase transition–a strategy similar to that of human papillomavirus (HPV), which also targets p21 or p16 [67–70]. Although herpesviruses share many common characteristics, they may have evolved specific strategies tailored to their replication. Our study demonstrates that HSV-2 regulates the cell cycle in a manner distinct from other human herpesviruses and is the first to reveal the molecular mechanism underlying HSV-2-mediated cell cycle progression in the context of virus infection. The CKI p21 (CDKN1A/Cip1/Waf1) is a critical cell cycle regulator [71]. In addition to this role, our proteomic data revealed several other cell cycle-related genes that are altered by HSV-2 infection. This suggests that viral manipulation of the host cell cycle involves a broad regulatory network, the elucidation of which will require systematic investigation in future studies, although it lies beyond the scope of this study.

The protein level of p21 was markedly reduced in infected cells, while its mRNA levels remained relatively unchanged, suggesting that HSV-2 regulates p21 through post-transcriptional or post-translational mechanisms. Given that p21 inhibits cyclin E–CDK2 activity, a key driver of G1/S transition [40–42], its downregulation is expected to promote S-phase entry. This hypothesis was supported by our rescue experiments, in which p21 overexpression reversed HSV-2-induced G1/S transition and suppressed viral replication. Beyond releasing the G1/S brake, the targeted degradation of p21 likely serves a broader strategic purpose for HSV-2. As an important regulator of DNA damage response, p21 mediates checkpoint enforcement [40]. Thus, its removal could not only facilitate cell cycle entry but also weaken the host’s ability to activate checkpoint-mediated defence against viral replication-induced stress.

We further demonstrate that HSV-2 not only promotes cell entry into the S-phase but also causes cell cycle arrest in this phase. HSV-2 downregulated the levels of CDK2 and its active phosphorylated form, while reducing the activity of cyclin A2–CDK2 complex, a key regulator of S-phase progression. While cyclin E–CDK2 functions primarily at the G1/S boundary to initiate S-phase entry, cyclin A2–CDK2 dominates the maintenance of S-phase progression and its arrest. Because our study focuses on sustained S-phase arrest and pseudo-S-phase formation following G1/S entry rather than the initial G1/S transition, we concentrated on characterizing the cyclin A2–CDK2 complex rather than the cyclin E–CDK2 complex. We found that HSV-2 infection markedly reduced p21 protein abundance, accompanied by a decreased G1-phase population and a robust increase in S-phase proportion, indicating an enhanced G1/S transition. These phenotypic changes suggest that the inhibition of cyclin E–CDK2 is relieved following removal of p21.

Proteomic analysis revealed broad downregulation of DNA replication complexes, including DNA polymerases, helicases and clamp-loading complexes. This coordinated disruption of the host DNA replication machinery may drive cells into a resource-rich yet replication-incompetent state termed ‘pseudo-S-phase’, in which cells display partial S-phase molecular features but remain incapable of genomic DNA replication. By arresting cells at the S-phase and preventing progression into mitosis, HSV-2 likely prolongs the S-phase, thereby providing sufficient time and cellular resources to support efficient viral DNA synthesis and replication. Of note, although proteomic analysis revealed the disruption of the host DNA replication machinery, future work is needed to determine whether host genomic DNA replication is indeed blocked.

Previous studies showed that sustained E2F3 activity is essential for the stable expression of replication machinery [72–74]. Intriguingly, E2F3 levels appeared to be reduced in HeLa cells following HSV-2 infection (Fig. S3A), whereas they remained unchanged in HFF-1 cells (Fig. S3B). It is known that HSV-2 encodes its own nucleotide metabolism enzymes, such as thymidine kinase and ribonucleotide reductase. The virus can directly activate the expression of both viral and host enzymes through its own viral proteins [19, 75]. Furthermore, cell cycle arrest at the S/G₂ phase has been shown to suppress the expression of interferon-stimulated genes and pro-apoptotic pathways [76, 77], thereby creating a permissive environment for viral replication. Our study establishes that S-phase accumulation enhances HSV-2 replication. Future investigations are needed to precisely delineate how HSV-2 exploits the S-phase environment to favour its replication. This biphasic hijacking – forcing entry into and then imposing arrest within a specific cell cycle phase – represents a profound disruption of cellular homeostasis. It underscores how pathogens can precisely reprogram essential cellular programmes to achieve a pathogen-favourable state, a concept that extends beyond virology to broader insights into cell fate decisions.

In this study, we assessed the effect of HSV-2 infection on cell cycle across different cell types, including transformed HeLa and untransformed cell lines (HFF-1 and ARPE-19). Consistent S-phase accumulation was observed across all tested cells, demonstrating that HSV-2-induced cell cycle reprogramming is a conserved and universal mechanism rather than a cell-type-specific phenomenon. Of interest, cyclin A2 abundance was markedly reduced in HFF-1 and ARPE-19 cells, though not in HeLa cells, whereas E2F3 levels decreased in HeLa cells but remained unaffected in HFF-1 cells. This differential regulation may be related to differences in cell state, as transformed cancer cells may rely less on cyclin A2 downregulation for cell cycle control. Additionally, HeLa cells possess defective p53 and Rb pathways due to HPV integration, whereas HFF-1 and ARPE-19 maintain intact cell cycle checkpoints. Future studies are needed to clarify the underlying mechanisms, whether E2F3 regulation is cell-type specific, involves other E2F family members and contributes to the HSV-2-mediated inhibition of host DNA replication. Of importance, HSV-2 consistently induced p21 degradation, accelerated G1/S transition, decreased CDK2 and its active phosphorylated form and ultimately enforced a robust S-phase arrest favourable to viral replication. The biphasic cell cycle hijacking is induced by HSV-2 regardless of the host cell’s baseline checkpoint integrity, revealing the significance and conservation of this viral replication strategy.

Our previous study suggested that the HSV-2 late protein UL24 likely contributes to S-phase accumulation, a finding that is further validated in this study (Fig. S4A-D). Our screening of immediate-early proteins revealed that ICP0 (RL2), ICP4 (RS1) and ICP22 (US1) also promote S-phase accumulation (Fig. S5A-C), suggesting that multiple viral factors likely work together to orchestrate S-phase accumulation. This functional redundancy mirrors findings from HSV-1, where ICP0, ICP4 and ICP22 collectively contribute to the disruption of cell cycle regulation [26, 78, 79]. As our study primarily focused on immediate-early proteins, future research should systematically characterize the roles of additional HSV-2 proteins to fully elucidate this coordinated viral network. Such efforts will be crucial for advancing our understanding of HSV-2 pathogenesis and guiding the development of multi-targeted antiviral strategies.

Using synchronized African green monkey kidney cells (CV-1) as a model system, a previous study by Hossain and colleagues provided early evidence that HSV-2 infection is associated with Rb phosphorylation and alterations in CDK2 activity [80]. However, the underlying mechanisms and functional significance for viral replication remain unclear. In the current study, we identify p21 degradation as the mechanistic trigger of G1/S transition and reveal that HSV-2 employs a biphasic strategy by first promoting S-phase entry and subsequently enforcing S-phase arrest to enhance viral replication. We further identify multiple viral proteins that cooperate in this process. Importantly, these findings were obtained under asynchronous culture conditions in HeLa, untransformed HFF-1 and retinal pigment epithelial (ARPE-19) cells, which more closely reflect physiological infection contexts than the serum-starved CV-1 model used previously. Together, our results demonstrate that HSV-2 intrinsically and robustly reprograms the host cell cycle to support viral replication without requiring external synchronization.

Based on our findings, we propose a model in which HSV-2 infection manipulates the host cell cycle to enhance viral replication (Fig. 6). Different cyclins exhibit distinct expression and degradation patterns, which contribute to the temporal coordination of cell cycle events. After HSV-2 infection, it expresses several viral proteins that collectively induce the degradation of p21 via the proteasome-mediated signalling pathway. The removal of a central G1/S checkpoint brake facilitates subsequent S-phase entry. Meanwhile, HSV-2 downregulates the levels of CDK2 and its active form, phosphorylated CDK2, as well as the activity of the cyclin A2–CDK2 complex, resulting in cells being arrested in S-phase. Ultimately, HSV-2 creates a ‘pseudo-S-phase’ environment that efficiently supports viral replication.

**Fig. 6.**
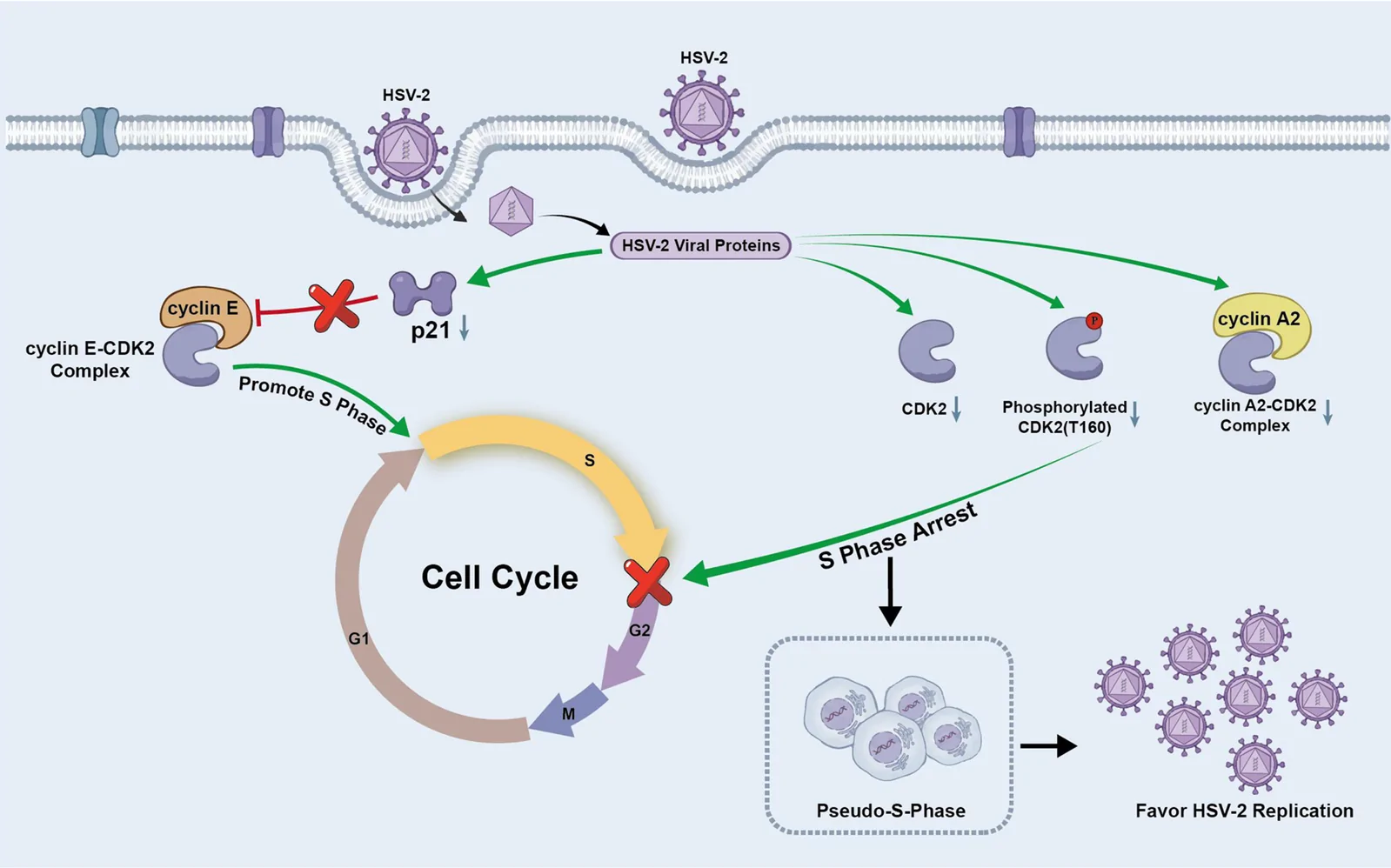
Schematic model of the mechanism by which HSV-2 promotes the accumulation of S-phase.

In conclusion, our study demonstrates that HSV-2 infection promotes S-phase entry by driving the proteasome-mediated degradation of p21, yet induces cell arrest in S-phase, thereby creating a favourable ‘pseudo-S-phase’ environment that enhances viral replication efficiency. These findings uncover a dual mechanism of host cell cycle subversion by a human pathogen, thereby enhancing our understanding of how pathogens disrupt cellular homeostasis to promote their replication.

## Supplementary material

10.1099/jgv.0.002346 Supplementary Material 1.

## Abbreviations

Abs: antibodies
CDK2: cyclin-dependent kinase 2
CDKN1A: cyclin-dependent kinase inhibitor 1A
CDKs: cyclin-dependent kinases
CKI: cyclin-dependent kinase inhibitor
DIA: data-independent acquisition
DMEM: Dulbecco's Modified Eagle Medium
FDR: false discovery rate
G1: gap 1
G2: gap 2
HeLa: human cervical epithelial cell
HFF-1: human foreskin fibroblast
hpi: hours post-infection
HPV: human papillomavirus
HSV-1: herpes simplex virus type 1
HSV-2: herpes simplex virus type 2
M: mitosis
qRT-PCR: quantitative real-time PCR
RPE: retinal pigment epithelia
S: synthesis
UPS: ubiquitin-proteasome system
