# Supplementary material for: Herpes simplex virus type 2 induces S-phase entry and arrest to regulate the cell cycle for efficient replication
Source: J Gen Virol. 2026 Oct 7;107(10):002346. doi: 10.1099/jgv.0.002346 (PMC13645362; doi:10.1099/jgv.0.002346)
Supplement: Supplementary file 1 [file jgv-107-02346-s001.pdf]

Supplementary Materials

HSV-2 induces S-phase entry and arrest to regulate the cell cycle for efficient replication

Huimin Wang[1], Ranqing Cheng[2, 3], Miaomiao Li[2, 3], Yuncheng Li[2, 3], Siyu He[2, 3], Longchao Xu[2, 3], Yuhao Chen[2, 3], Zhenhua Huang[2, 3], Binlian Sun[1], Yalan Liu[2, 3 *], Qinxue Hu[2, 3 *], Mudan Zhang[2, 3 *]

[1] Hubei Key Laboratory of Cognitive and Affective Disorders, Institute of Biomedical Sciences, School of Medicine, Jianghan University, Wuhan 430056, China

[2] State Key Laboratory of Virology and Biosafety, Wuhan Institute of Virology, Chinese Academy of Sciences, Wuhan 430071, China

[3] Medical School, University of Chinese Academy of Sciences, Beijing 100049, China

*Correspondence: E-mail: mudan@wh.iov.cn; Tel.: +86 27 87998706 (MZ, https://orcid.org/0000-0002-5164-5891). E-mail: qhu@wh.iov.cn; Tel.: +86 27 87998706 (QH).

Table S1. Primers used in this study

Primer Name	Sequence [a]	Cloning Sites
**For plasmid construction**		
p21-F	5'-TAGCGTTTAAACTT**AAGCTT**ATGTCAGAACCGGCTGGGGATGTCC-3'	Hind III
p21-R	5'-CTCGA**GCGGCCGC**CTACTTATCGTCGTCATCCTTGTAATCGGGCTTCCTCTTGGAGAAG-3'	Not I
**For real time PCR**		
CDKN1A-F	5'-TGTCCGTCAGAACCCATGC-3'	
CDKN1A-R	5'-AAAGTCGAAGTTCCATCGCTC-3'	
GAPDH-F	5'-AAGGTCGGAGTCAACGGATTTG-3'	
GAPDH-R	5'-CCATGGGTGGAATCATATTGGAA-3'	

[a] Restriction enzyme sites are highlighted in bold.
F, forward. R, reverse.

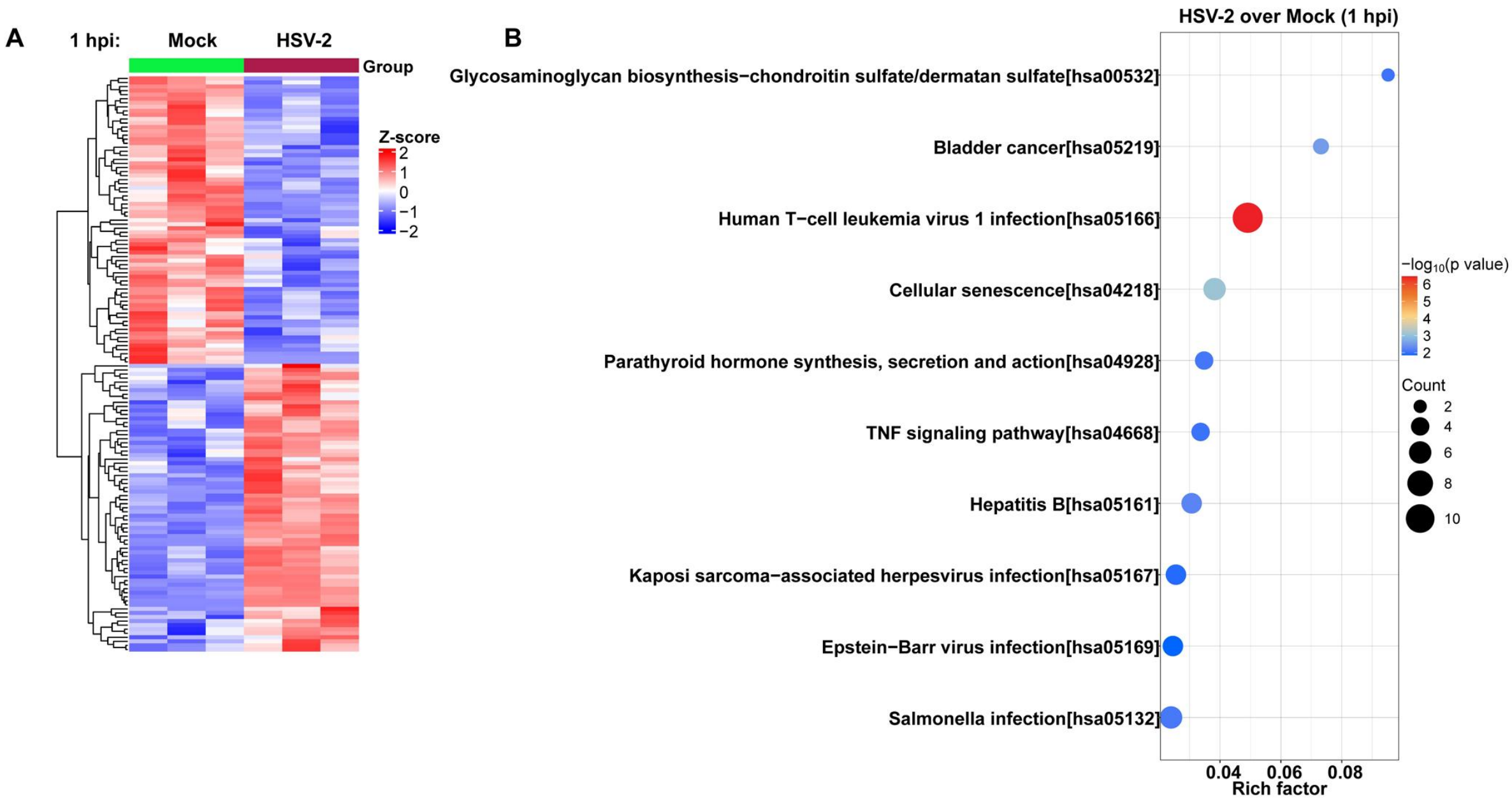

Fig S1. Proteomic profiles of HSV-2-infected HeLa cells. A. Hierarchical clustering of differentially expressed proteins in HeLa cells at 1 hpi with HSV-2. Each column represents an individual biological replicate, and each row represents a protein. Protein expression levels are normalized as Z-score (red, upregulated; blue, downregulated). B. KEGG pathway enrichment analysis of the differentially

expressed proteins at 1 hpi. Dot size indicates the number of proteins in each pathway, and dot color represents the $-log_{10}$ (p value).

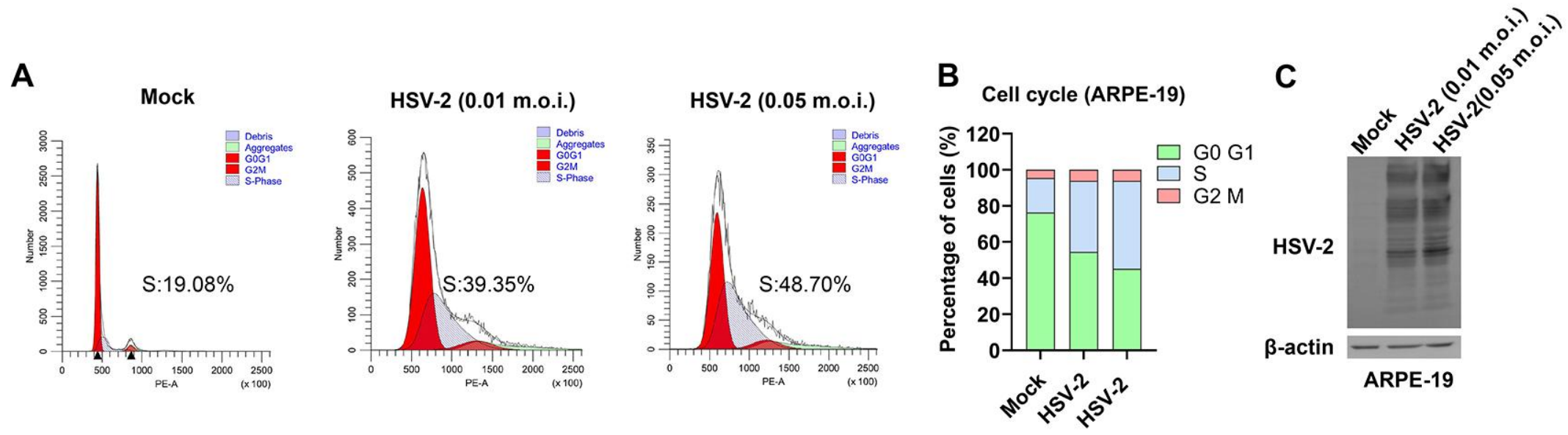

Fig S2. HSV-2 drives S-phase accumulation by enhancing G1/S transition. ARPE-19 cells were infected with HSV-2 at the indicated m.o.i.. At 24 hpi, cells were harvested for cell cycle distribution analysis by flow cytometry (A), or lysed for Western blot assay to confirm successful infection (C). The percentages of cells in each cell cycle phase were quantified and analyzed using GraphPad Prism 9 (B). Data shown are representative of three independent experiments.

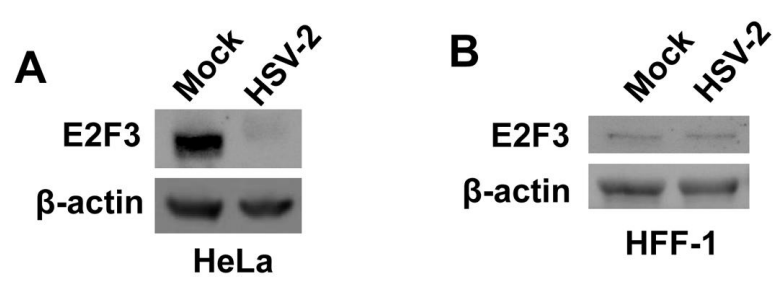

Fig S3. **The production of E2F3 following HSV-2 infection**. HeLa (A) and HFF-1 (B) cells were infected with HSV-2 (m.o.i. = 0.05). At 24 hpi, the protein level of E2F3 was assessed by Western blot using specific primary antibody. Data shown are representative of three independent experiments.

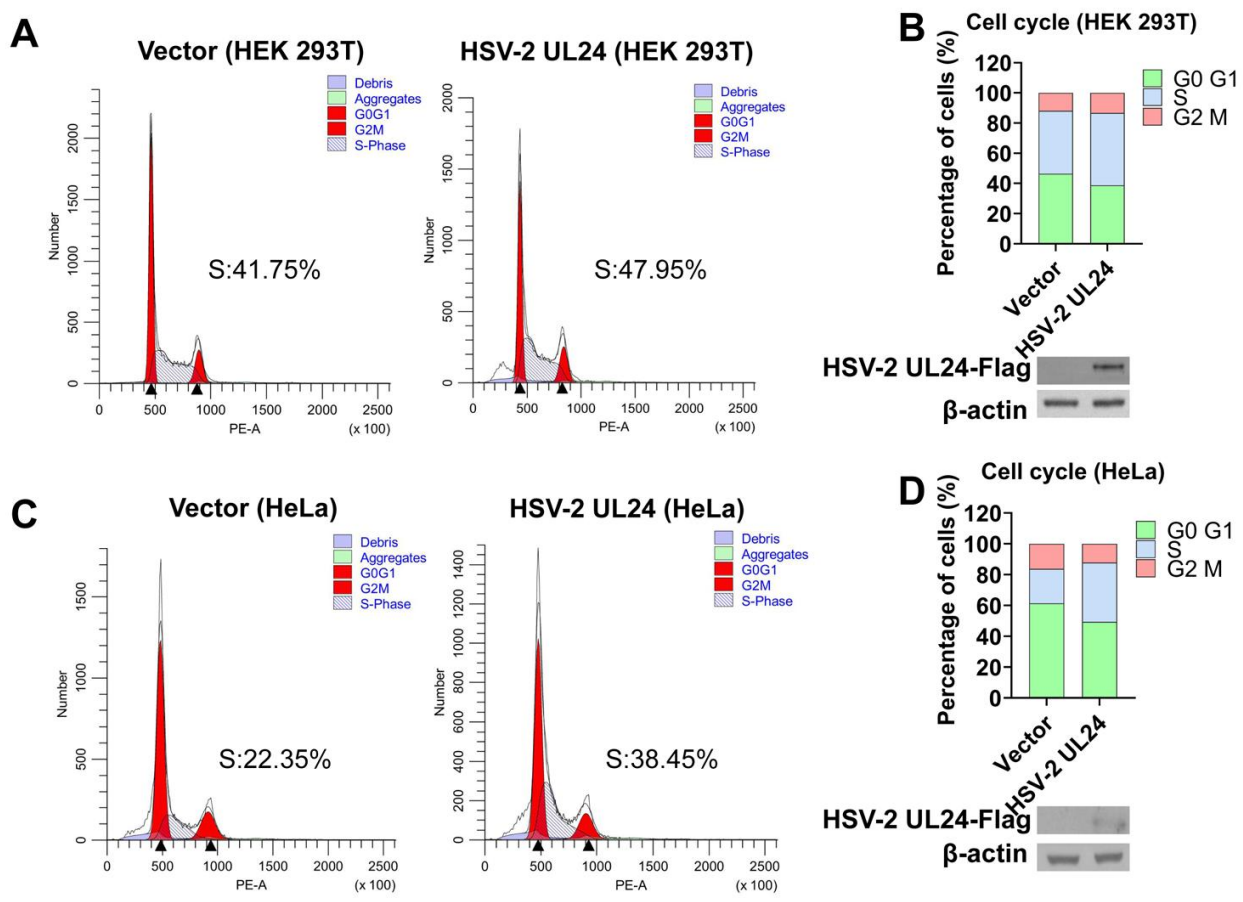

Fig S4. HSV-2 UL24 promotes S phase accumulation. HEK 293T cells (A and B) or HeLa cells (C and D) in 6-well plates were transfected with empty vector or UL24 expression plasmid for 48 hours. The cell cycle was analyzed by flow cytometry analysis (A and C). The percentages of cells in each cell cycle phase were quantified and analyzed using GraphPad Prism 9 (B and D). The expression of UL24 was confirmed by Western blot. Data shown are representative of three independent experiments.

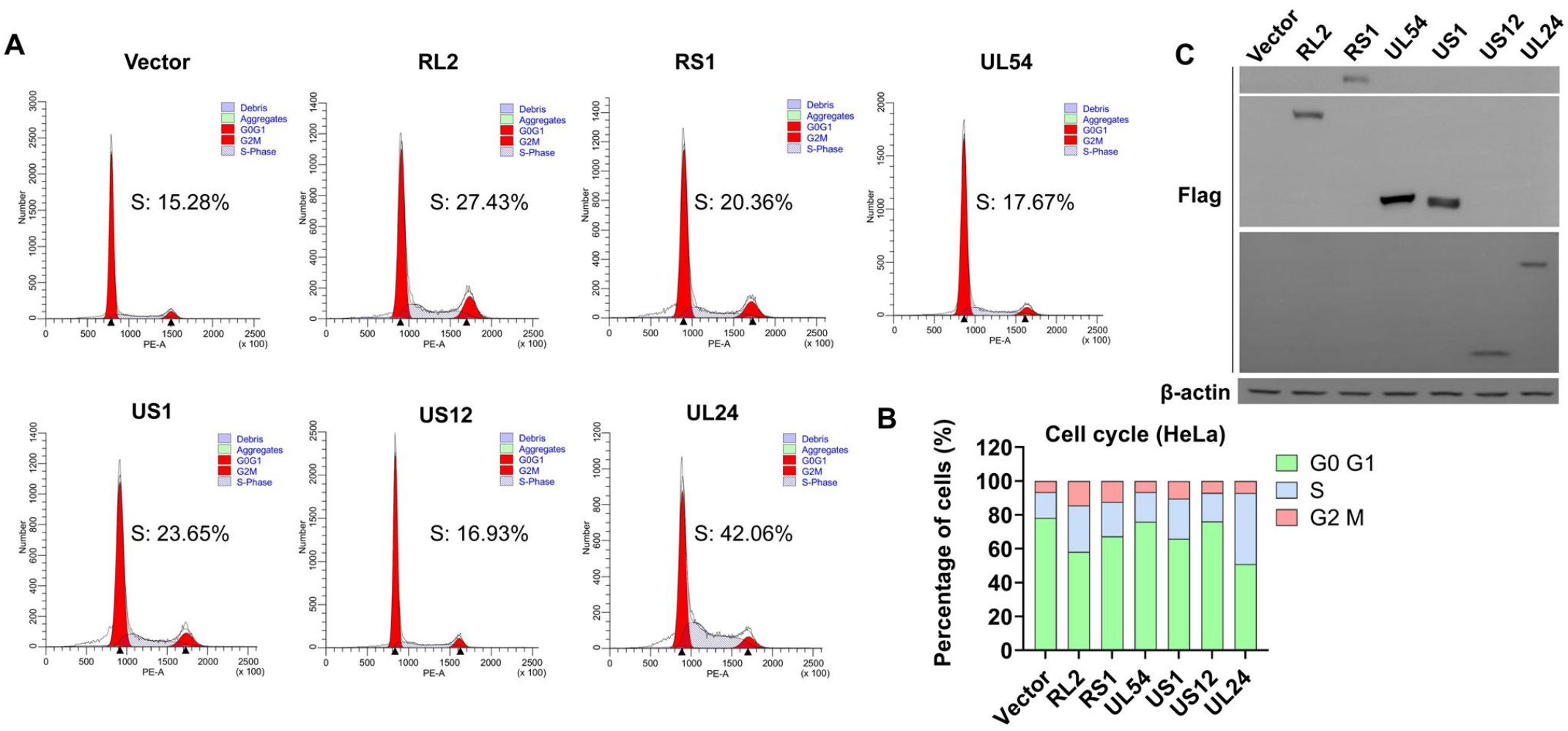

Fig S5. Multiple HSV-2 proteins regulate the progression of the S phase. HeLa cells in 6-well plates were transfected with empty vector or HSV-2 protein expression plasmid for 48 hours. The cell cycle was analyzed by flow cytometry analysis (A).

The percentages of cells in each cell cycle phase were quantified and analyzed using GraphPad Prism 9 (B). The expression of HSV-2 protein was confirmed by Western blot (C). Data shown are representative of three independent experiments.
